# Multimodal Treatment of Advanced Mucosal Melanoma in the Era of Modern Immunotherapy

**DOI:** 10.3390/cancers12113131

**Published:** 2020-10-26

**Authors:** Pawel Teterycz, Anna M. Czarnecka, Alice Indini, Mateusz J. Spałek, Alice Labianca, Pawel Rogala, Bożena Cybulska-Stopa, Pietro Quaglino, Umberto Ricardi, Serena Badellino, Anna Szumera-Ciećkiewicz, Slawomir Falkowski, Mario Mandala, Piotr Rutkowski

**Affiliations:** 1 Department of Soft Tissue/Bone Sarcoma and Melanoma, Maria Sklodowska-Curie National Research Institute of Oncology, 02-781 Warsaw, Poland; pawel.tetrycz@pib-nio.pl (P.T.); mateusz@spalek.co (M.J.S.); pan.rogal@gmail.com (P.R.); slaw.falkowski@gmail.com (S.F.); piotr.rutkowski@coi.pl (P.R.); 2 Melanoma Unit, Department of Oncology and Hematology, Papa Giovanni XXIII Hospital, 24127 Bergamo, Italy; alice.indini@gmail.com (A.I.); alabianca@asst-pg23.it (A.L.); mmandala@asst-pg23.it (M.M.); 3 Maria Skłodowska-Curie National Research Institute—Oncology Center, Krakow Branch, 31-115 Krakow, Poland; bcybulskastopa@vp.pl; 4 Department of Medical Sciences, Dermatologic Clinic, University of Turin, 10126 Turin, Italy; pietro.quaglino@unito.it; 5 Department of Oncology, Radiation Oncology, University of Turin, 10126 Turin, Italy; umberto.ricardi@unito.it; 6 Department of Oncology, Radiotherapy Unit, AOU Città della Salute e della Scienza di Torino, 10126 Turin, Italy; serena.badellino@tiscali.it; 7 Department of Pathology and Laboratory Medicine Maria Sklodowska-Curie Memorial Cancer Center and Institute of Oncology, 02-781 Warsaw, Poland; szumann@gmail.com; 8 Department of Diagnostic Hematology, Institute of Hematology and Transfusion Medicine, 02-776 Warsaw, Poland

**Keywords:** mucosal melanoma, nivolumab, pembrolizumab, ipilimumab, radiotherapy

## Abstract

**Simple Summary:**

Immunotherapy revolutionized the treatment of cutaneous melanoma and greatly improved treatment outcomes in this group of patients. Mucosal melanoma is a rare disease, biologically distinct from the cutaneous subtype. There is little real-world data on immunotherapy efficacy in mucosal melanoma. Therefore, we aimed to analyze and describe experiences in mucosal melanoma treatment in five high volume oncology centers in Europe. Furthermore, we evaluated if concomitant radiotherapy may improve the outcomes of these patients. We conclude that immunotherapy with anti-PD1 antibodies is a safe and effective treatment of mucosal melanoma. Concomitant radiotherapy may be beneficial in a selected subgroup of patients with advanced mucosal melanoma.

**Abstract:**

Mucosal melanoma is a rare disease epidemiologically and molecularly distinct from cutaneous melanoma developing from melanocytes located in mucosal membranes. Little is known about its therapy. In this paper, we aimed to evaluate the results of immunotherapy and radiotherapy in a group of patients with advanced mucosal melanoma, based on the experience of five high-volume centers in Poland and Italy. There were 82 patients (53 female, 29 male) included in this retrospective study. The median age in this group was 67.5 (IQR: 57.25–75.75). All patients received anti-PD1 or anti-CTLA4 antibodies in the first or second line of treatment. Twenty-three patients received radiotherapy during anti-PD1 treatment. In the first-line treatment, the median progression-free survival (PFS) reached six months in the anti-PD1 group, which was statistically better than 3.1 months in the other modalities group (*p* = 0.004). The median overall survival (OS) was 16.3 months (CI: 12.1–22.3) in the whole cohort. Patients who received radiotherapy (RT) during the anti-PD1 treatment had a median PFS of 8.9 months (CI: 7.4–NA), whereas patients treated with single-modality anti-PD1 therapy had a median PFS of 4.2 months (CI: 3.0–7.8); this difference was statistically significant (*p* = 0.047). Anti-PD1 antibodies are an effective treatment option in advanced mucosal melanoma (MM). The addition of RT may have been beneficial in the selected subgroup of mucosal melanoma patients.

## 1. Introduction

Mucosal melanoma (MM) is a rare disease epidemiologically and molecularly distinct from cutaneous melanoma (CM) developing from melanocytes located in mucosal membranes. Overall, MM represents about 1 to 1.5% of all melanoma cases and 0.03% of all diagnosed cancers [1,2,3]. MM incidence has been reported globally stable over the last 20 years [4,5]. MM is diagnosed twice more often in Caucasians than in populations with darker skin colors, including African Americans [6,7], but much rarer than in the Asian population. The risk of developing MM increases with age. The majority of patients diagnosed are 60 years of age and older. The median age at diagnosis is 70 years, except for MMs arising in the mouth that affect younger patients more frequently [6,8]. Anatomically, it is mostly diagnosed on the mucous membranes of the head and neck (31% to 55%), anus and rectum (17% to 24%) or the vulva and vagina (18% to 40%) and less frequently in the colon, throat, larynx, lungs, urinary tract, cervix, esophagus or gallbladder [3,8,9]. The incidence of MM is over 80% higher in women than in men due to the relatively high number of genital melanomas diagnosed in women [3]. Risk factors for developing MM are currently unknown since ultraviolet (UV) radiation and viral etiology—cytomegalovirus, Eppstein-Barr virus, human papillomavirus, human herpesvirus—have also been excluded. In MM, no environmental exposures nor carcinogenic viruses have been found pathogenic [10,11,12,13,14]. Unlike cutaneous melanoma, which is characterized by a UV signature, MM harbors distinct molecular features, including a lower incidence of v-Raf murine sarcoma viral oncogene homolog B **(***BRAF*) oncogene mutations but a higher incidence of tyrosine-protein kinase KIT (CD117) oncogene mutations, suggesting different genetic etiologies. In general, MM harbors fewer nucleotide substitutions per cell than CM, but more gene amplifications and structural variants than CM; the cause of this chromosomal instability has not yet been clarified [3].

Mucosal melanomas are generally diagnosed in an advanced stage, though they are more aggressive and carry a worse prognosis regardless of the stage at diagnosis. The five-year overall survival (OS) rate for MM is only 25% regardless of stage [15]. Poor treatment results and shorter survival may be associated with a lack of early symptoms or signs, resulting in advanced disease at the time of diagnosis. Insidious anatomical localization, and often amelanotic presentation, result in difficult visual detection and challenging resections with wide, or even negative, margins impossible to achieve. Moreover, rich lymphatic drainage from the mucosal surfaces promote metastases [3,8,15]. MM metastases most often develop in the lungs (54%), liver (35%) and bone (25%) [16].

Due to its rare occurrence, undefined etiopathogenesis and unpredictable clinical course, there are no specific recommendations of MM treatment. Both the European Society for Medical Oncology (ESMO) and National Comprehensive Cancer Network (NCCN) guidelines focus on the important role of surgery and radiotherapy (RT) in this group of patients [2,17]. The preferred therapeutic strategy in MM is still surgical excision. Perioperative RT improves local control but does not improve overall survival (OS), probably because of the high rate of distant relapse [18,19,20]. RT with definitive intent can also provide satisfactory local control. Definitive RT should be considered in patients who are not candidates for extensive surgery or in cases where adequate resection margin cannot be achieved [21]. Proton therapy and heavy ions deserve special attention [22]. In a retrospective analysis of a cohort of patients with sinonasal MM, the authors observed a 62% five-year local control rate for proton therapy [23]. The understanding of multimodal treatment in MM comes mostly from CM data extrapolation. Phase III clinical trials focused only on MM patients are lacking. Phase III trials with all melanoma patients, regardless of subtype, do not provide satisfactory data on MM due to the low number of cases enrolled. The real-world data on the efficacy of antiprogram med cell death 1 (PD-1) therapies in patients with MM are scarce. Although a growing number of studies suggest the significant benefit of RT as a boost for immunotherapy (ITH) in CM, such observations in MM have not been reported. Therefore, this study aims to assess the efficacy of systemic therapy with the emphasis on ITH in the MM patient population treated outside of clinical trials, and to define the efficacy of the immunotherapy-radiotherapy combination in MM.

## 2. Results

### 2.1. Cohort

Eighty-two patients from the participating centers who met inclusion criteria were enrolled. The clinicopathological characteristics of patients and tumors are summarized in Table 1. The median follow-up, as estimated by the reverse Kaplan-Meier method, was 29.0 months (CI: 19.1–41.7). At the time of data analysis—August 2020, 30 (37%) patients remained alive on the treatment or in follow up. It is worth mentioning that in fourteen (17.1%) cases, the systemic treatment was initiated due to unresectable tumors without distant metastases (with the majority of cases in the head and neck region—ten). MM, located in the genitourinary system, was predominantly present in females (female to male ratio = 21:1); no such differences were observed in any other location. The BRAF V600 mutation was detected in 4/33 patients from the head and neck region and in 1/49 patients in other locations (rectum).

### 2.2. Treatment

The summary of administered systemic treatment is shown in Table 1. Most of the patients received anti-PD1 ITH in the first line (*n* = 63, 76.8%). Progression of the disease on the first-line treatment was observed in 63 patients, including 44 treated with anti-PD1 ITH. After progression of the disease (PD), forty-three patients (68%) were eligible for second-line therapy. Among those patients, 21 received ipilimumab, while 15 patients received anti-PD1 ITH antibodies. Overall, only five patients did not undergo anti-PD1 treatment.

### 2.3. Response

In the first-line treatment, OR and disease control (DC) were observed in 16 (25%) and 37 (59%) patients in the anti-PD1 ITH group (*n* = 63); and 3 (15%) and 7 (37%) patients in the other treatments group (*n* = 19), respectively. In the second and subsequent treatment lines, OR lasting longer than one year was observed only in two patients in the ipilimumab group (*n* = 21), and in four patients treated with anti-PD1 ITH (*n* = 15).

**Figure 1:**
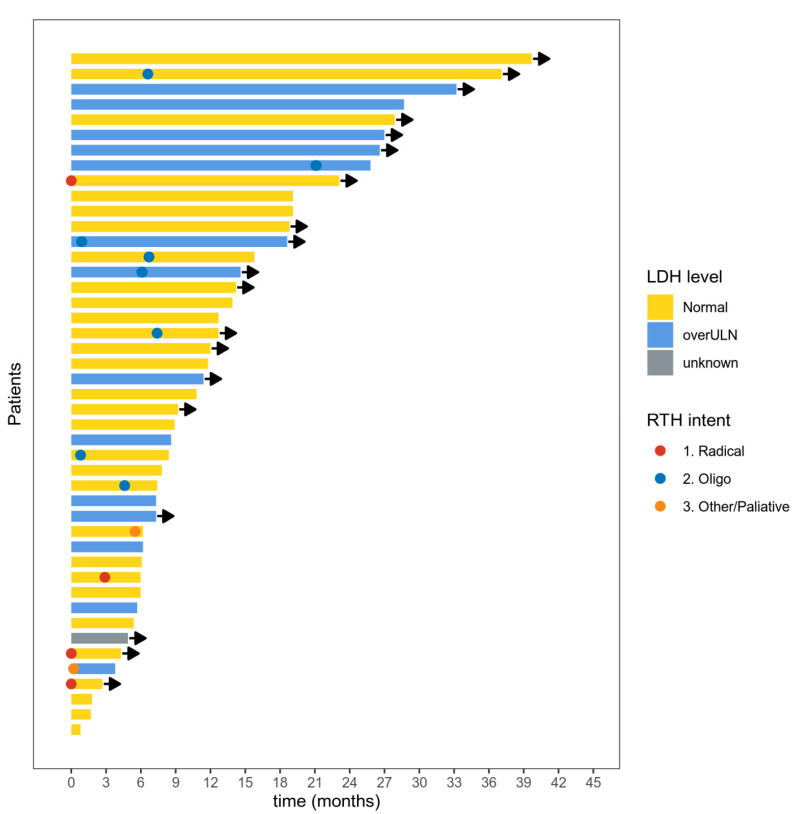
Swimmer plot for the duration of response to the anti-PD1 immunotherapy. Each bar represents a case; arrows represent an ongoing response. LDH level = lactic dehydrogenase activity at the start of treatment. overULN = over upper limit normal. RTH intent = intention of radiotherapy. Oligo = radiotherapy due to oligometastatic/oligoprogressive disease. Radiotherapy after progressive disease according to Response Evaluation Criteria in Solid Tumours (RECIST) 1.1 is not shown.

In the second and subsequent treatment lines, DC was observed in six patients in the ipilimumab group (*n* = 21) in eight patients in the anti-PD1 ITH group (*n* = 15), two patients treated with dacarbazine-based chemotherapy and one treated with imatinib. The duration of response to anti-PD1 ITH has been visualized as a swimmer plot in Figure 1.

### 2.4. Progression-Free Survival

**Figure 2:**
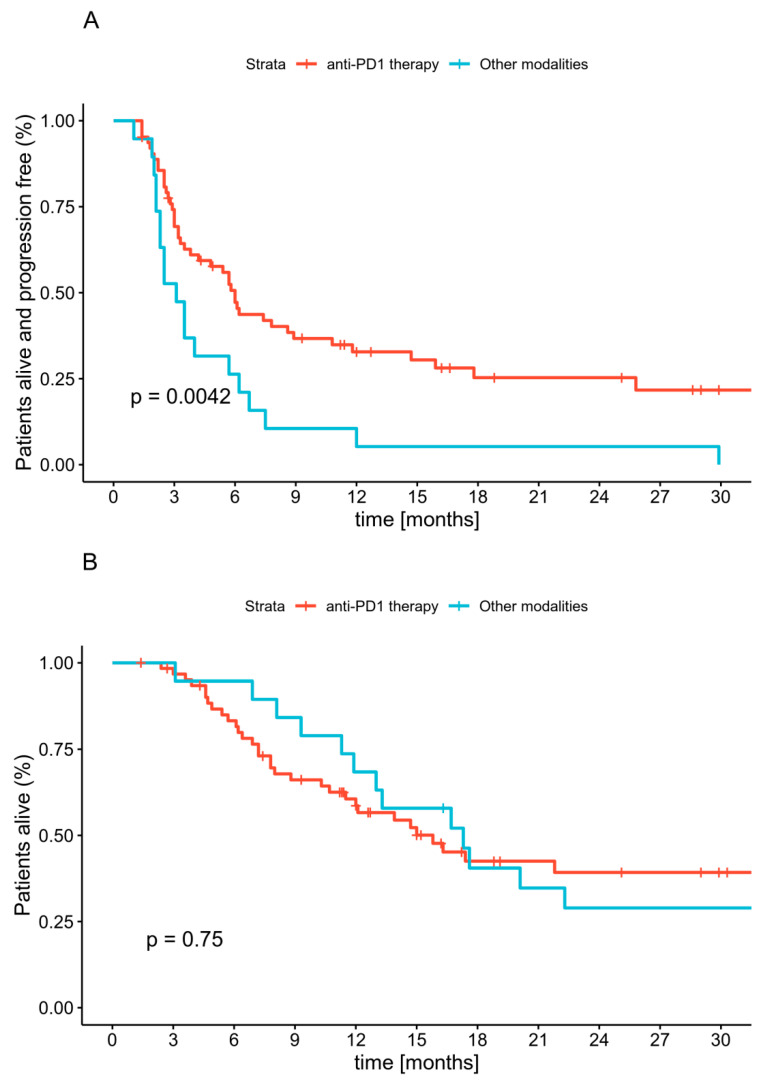
Progression-free survival (**A**) and overall survival (**B**) in mucosal melanoma patients in subgroups treated with anti-PD1 therapy (red curve) or other modality (blue curve) in first-line.

In the first-line treatment, the median progression-free survival (PFS), 12-month and 18-month PFS rate reached six months (CI: 3.8–10.8), 33% (CI: 23–48%) and 25% (CI: 16–41%), respectively, in the anti-PD1 ITH group. The same parameters were equal to 3.1 months (CI: 2.3–6.7), 5% (CI: 1–36%) and 5% (CI: 1–36%), respectively, in the other treatments group. These differences were statistically significant by the log-rank test with *p* = 0.004 (Figure 2A). Long-term disease stabilization was not achieved in patients treated with other systemic treatments. None of the preselected prognostic factors had a significant influence on PFS. Patients who received treatment other than anti-PD1 ITH had HR 2.18 (CI: 1.26–3.76, *p* = 0.005) (Table 2) of progression.

In the second line of treatment, the median PFS reached 6.2 months (CI: 2.8–NA) for anti-PD1 ITH, 3.0 months (CI: 2.6–10) for ipilimumab and 4.6 months (CI: 1.8–NA) for other treatments. These differences were not statistically significant (*p* = 0.51).

### 2.5. Overall Survival

The median OS was 16.3 months (CI: 12.1–22.3) in the whole cohort. The 12-month and 18-month OS rates reached 61% (CI: 51–73%) and 41% (CI: 31–55%), respectively. There was no OS difference between patients who received anti-PD1 ITH or other treatments as first-line therapy (*p* = 0.7). Kaplan-Meier curves for OS are presented in Figure 2B. Men and patients with elevated LDH levels had a worse prognosis. None of the other preselected clinical factors had a significant influence on OS in this group of patients (Table 2).

### 2.6. Radiotherapy

**Figure 3:**
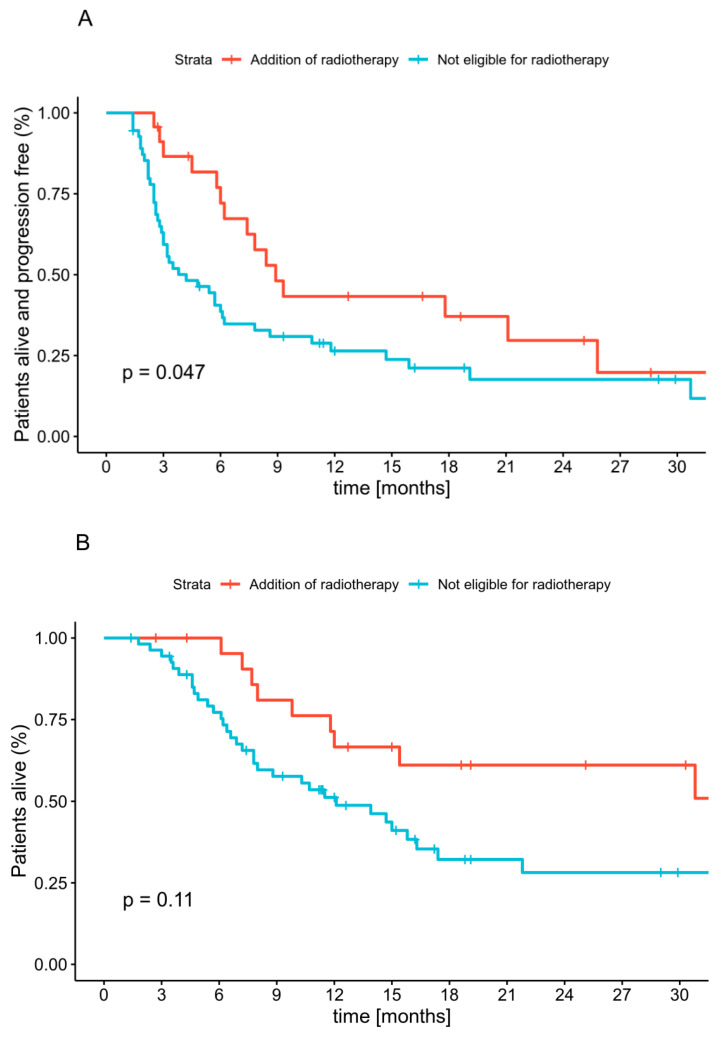
Progression-free survival (**A**) and overall survival (**B**) since the start of anti-PD1 therapy according to radiotherapy use.

Twenty-three patients received RT during anti-PD1 ITH (Supplement A). The one-year local control rate (LCR) in the subgroup who received RT with anti-PD1 ITH for unresectable locally advanced disease reached 75.0% (CI: 42.6–100%). This value was stable throughout the two years of follow-up. The one-year LCR after RT for the oligometastatic disease during anti-PD1 ITH was 85.7% (CI: 63.3–100%). Patients who received RT during the anti-PD1 treatment had a median PFS of 8.9 months (CI: 7.4–NA), whereas in patients who received anti-PD1 ITH as the only treatment the PFS was 4.2 months (CI: 3.0–7.8); the difference was statistically significant (HR 1.8. CI: 1.0–3.3, *p* = 0.05). The median OS since the start of anti-PD1 ITH was 32.2 months (12.0–NA) in patients who received RT, and 12.1 months (CI: 7.8–21.8) in nonirradiated patients. This was not statistically significant (*p* = 0.11) (Figure 3, Supplement A ).

## 3. Discussion

Until now, limited evidence has supported the efficacy of anti-PD-1 ITH in MM. In this population of patients, in individuals with the KIT mutation, imatinib was reported to result in significant clinical benefits, while for MM patients whose tumors harbor *BRAF* mutations, treatment with a combination of BRAF and MEK inhibitors was shown to be effective [8,24]. The efficacy of anti-PD1 ITH remains unclear in MM patients since a low number of patients have been enrolled in clinical trials. Conflicting data on MM patients’ responses to nivolumab, pembrolizumab and ipilimumab have been published. Some reports suggest that anti-PD ITH is significantly less effective in MM than in CM patients, while other report durable anti-tumor effects [25,26,27].

We analyzed anti-PD-1 ITH efficacy in routine clinical practice outside of clinical trials in a nonpreselected population of subsequent patients with MM, and report ORR and PFS similar to patients with CM, also treated outside of clinical trials. In a retrospective analysis covering 25 dermatology departments in France, 75 MM patients were treated with first-line nivolumab or pembrolizumab and, out of these, fifteen achieved OR, which corresponds to an ORR of 20% (95% CI: 11.6–30.8) [28], which is lower than the 25% reported by us. In a Japanese study of 24 MM cases, the ORR was 20.8%, and for 17 cases with visceral metastases—17.6%. For the 17 cases, OS and PFS periods were 422 days and 226 days [29]. In another small Japanese trial, 17 MM patients were treated with nivolumab, and ORR was reported at 23.5%. One patient achieved a CR, three—PR, and five SD as their best response. The median PFS was 1.4 months (95% CI: 1.2–2.8) [30], which is again inferior to the results reported by us. High OR was reported for the first-line pembrolizumab—35% accompanied with five-months of median PFS (*n* = 20) [31].

We previously described the results of anti-PD1 therapy as well as BRAF/MEK inhibitors treatment in the general melanoma population in Poland [32]. While comparing data between the general population of patients receiving anti-PD1 ITH in the first line and an analogous subpopulation of mucosal melanoma patients, no differences were seen in ORR (objective response rate), PFS and OS. The ORR was equal to 28% in the mucosal melanoma subgroup, and 30% in the general population (*p* = 0.85 by Fisher’s exact test). The median PFS and OS for mucosal melanoma was 6.0 (CI: 3.8–10.8) and 15.8 (CI: 11.5–NA) months, while for the general population—6.9 (CI: 5.3–9.0) and 20.5 (CI: 15.3–NA), respectively. The HR for mucosal melanoma was 0.97 (CI: 0.68–1.38, *p* = 0.86) for PFS and 0.91 (CI: 0.60–1.38, *p* = 0.65) for OS (see Supplement B). Therefore, our results confirm the efficacy of immunotherapy, especially anti-PD1 antibodies, in MM treatment [31].

Due to strict inclusion criteria in clinical trials, covering mostly patients without brain metastases and patients with good performance status, corresponding to low tumor burden, we expected longer PFS and OS for patients treated in the trials than for patients treated in our study. In a meta-analysis of major ITH clinical trials by Shoushtari, A.N. et al., 35 MM patients were identified. This analysis covered patients treated within NCT02083484 (MK-3475), NCT01295827 (KEYNOTE-001), NCT01927419 (CheckMate 069), NCT01024231 (CA209-004), and NCT01721746 (CheckMate 037) trials. Therefore, the majority of patients were treated with pembrolizumab or nivolumab, not in the first line but after previous therapy. In these trials the majority of patients received ipilimumab before anti-PD-1 ITH. These authors reported that for MM treated with anti-PD-1 ORR was 23% (95% CI: 10–40%) with PD as the best response for 57% of patients (95% CI: 39–74%), which represents ORR similar as that reported by us (25% for anti-PD1 in the first line). In the same meta-analysis, MM patients treated with nivolumab/pembrolizumab monotherapy achieved PFS of 3.9 months, which is shorter than the PFS of our patients treated in the first line. ORR reported by Shoushtari, A.N. et al. is also generally numerically similar to the 23% ORR reported by D’Angelo, S.P *et al.* for 86 MM patients included in another meta-analysis of multiple prospective trials of nivolumab; but seven patients—that is almost 10% of analyzed cases—were included in both described analyses, and these data may not be interpreted independently [25,33]. In the analysis by D’Angelo, S.P et al. covering, again, mostly patients treated with anti-PD1 ITH in further lines of therapy in NCT00730639 (CA209-003), NCT01621490 (CA209-038), NCT01721772 (CheckMate 066), NCT01721746 (CheckMate 037), and NCT01844505 (CheckMate 067) trials, ORR was—37.1% (95% CI, 21.5% to 55.1%) and the median PFS was 3.0 months (95% CI, 2.2 to 5.4 months), which represents higher ORR, but shorter PFS than reported by us [25]. For pembrolizumab only trials—KEYNOTE-001 (NCT01295827), -002 (NCT01704287), and -006 (NCT01866319), the ORR was 22% (95% CI: 11–35) and 15% (95% CI: 5–32) in ipilimumab-naive and ipilimumab-treated MM patients, which is concordant with our treatment results. At the same time, the median PFS was 2.8 months (95% CI: 2.7–2.8) for KEYNOTE-001/-002/-006 patients, which is less than half that of the six months reported by us. Similar ITH efficacy was reported in eight patients pretreated with dacarbazine before nivolumab administration [34]. The administration of nivolumab was listed as long-term for three patients with 13–17 cycles given, and over 30 cycles for one case. In this group, ORR was 37.5% (CR—25.0%, PR—12.5%), median PFS—10.2 months [35].

It should be noted that in the analysis by D’Angelo, S.P et al., median PFS in patients treated with nivolumab combined with ipilimumab (*n* = 35) was 5.9 months (95% CI: 2.8 months—not reached), while our patients (*n* = 63) achieved median PFS of six months during anti-PD-1 monotherapy therapy. In concordance with our report, in the pooled analysis CM patients achieved PFS of 6.2 months with nivolumab monotherapy and 11.7 months with nivolumab-ipilimumab combination therapy [25]. Nevertheless, in the pooled analysis of major clinical trials, MM patients achieved reduced clinical benefits in comparison to CM patients during anti-PD-1 ITH treatment [25]. This was not the case in routine clinical practice reported by us with PFS in a general population of 6.9 months and OS of 20.5 and resultant HR for MM of 0.95 for PFS and 0.89 for OS. On the contrary, in clinical trial data, MM patients who were treated with nivolumab monotherapy received a median of 7.0 doses (1–34) and CM patients 11.0 doses (1–61) [25]. Moreover, in MM patients, the median reduction in tumor burden in the target lesions was reported only at 1.4% for nivolumab monotherapy and as high as 34.2% for combined therapy [25], which may indicate the specific biology of the disease requiring dual pathway activation for MM cell elimination.

In terms of OS of MM, patients treated with ITH were reported to achieve a median OS of 15.97 months and a one-year OS rate of 57.8% (95% CI: 49.5–67.5) while no specific data for nivolumab, pembrolizumab and ipilimumab differences were reported [28]. In a Japanese trial, median OS was 12.0 months (95% CI: 3.5—not reached) for nivolumab treatment, while for pembrolizumab pooled analysis the median OS was 11.3 months (7.7–16.6) [30,34]. In general, despite the limitations of the studies discussed, data suggest that in MM patients anti-PD1 therapy may be effective both in terms of OR, PFS and OS. Nevertheless, it should be considered that in all of the multi-trial analyses, the number of MM cases was about 10% of CM patients, which may influence statistical calculations [25]. With our report, we confirm the efficacy of ITH in MM patients with real-world data.

The efficacy of ITH may be enhanced by RT, which was reported before (ORR = 57.1%) in the case of seven patients [36]. Another analysis of 10 patients covered mucosal melanoma of the nasal cavity or maxillary sinus, in which patients were treated with nivolumab or pembrolizumab and concomitant radiotherapy. In this group of patients, after a median follow-up period of 46 weeks, the local control rate of the primary lesion and regional lymph nodes was 100% with a median PFS of 7.4 months (range 2–82 weeks). In this group of patients, the six-month PFS rate was 60% [37], which is lower than in our study—8.9 months. The largest report published until now was a retrospective study of 23 patients out of whom 12 patients were treated with pembrolizumab and RT, 11 patients were treated with RT alone and the others were treated with pembrolizumab monotherapy. It was shown that pembrolizumab with concomitant radiotherapy enabled achievement of a one-year target lesion control rate in 94.1% cases, while radiotherapy without immunotherapy enabled the control in 57.1% and pembrolizumab in 25%. Treatment-related AEs were not significantly different between radiotherapy with or without pembrolizumab [38]. In our study all 23 patients received concomitant RT and immunotherapy (Supplement C). Generally, concomitant RT is not allowed in the majority of clinical trials. Thus, a separate study with RT-ITH is required to confirm the expected benefit of irradiation during ITH. While we report the data, a separate trial is also ongoing (NCT04017897).

At the same time, no significant association is known between PFS duration and other clinical factors including primary tumor localization, *BRAF* gene mutation status, stage at treatment start, presence of CNS or liver metastases, type of prior therapy, or response to first-line (ipilimumab) therapy if used in the second-line, as in the case of our analysis (Table 2) [31,33]. The duration of OS and PFS are correlated with irAE development and high levels of PD-L1 expression (>5% of cells in the tumors) [37,38]. In fact in patients with mucosal melanoma and tumor PD-L1 expression ≥ 5% (n = 15), ORR was 53.3% (95% CI: 26.6–78.7%), while in patients with PD-L1 expression < 5% (n = 49)—ORR is 12.2% (95% CI: 4.6–24.8%), and in these patients median PFS with PD-L1 expression ≥ 5% was 12.2 months (95% CI: 3.0 months—not reached), while for PD-L1 expression < 5%—median PFS was < 3 months while the role of tumor PD-L1 expression as a response biomarker was not fully defined [25,39]. In fact, the significantly lower mutational burden in MM in comparison to CM may explain the decreased efficacy of the immune checkpoint blockade in many MM patients [40,41]. In general, not only PD-1/PD-L1 expression level, but also immune cells infiltration and transcriptional immunoscore, may be correlated with immunotherapy response. Multiple genetic and genomic factors, including mutation burden, mismatch repair deficiency, somatic copy-number variation burden and neoantigen load are being suggested to predict immunotherapy response [42]. While somatic mutation clonality was reported to positively correlate with immunotherapy response, copy number variation (CNV) was shown to negatively correlate with efficacy of response to the PD-1 checkpoint blockade and to be associated with downregulation of immune-related pathways expression [43,44]. Moreover, the expression of melanin pigment and active melanogenesis, which are often found at high levels in MM, may decrease the sensitivity of MM cells to immunotherapy [45,46]. Melanin also has radioprotective and scavenging properties and may decrease the efficacy of radiotherapy [47]. The relationship had not been finally defined as in survival meta-analysis pigmentation; that is, melanin level was not significantly correlated with survival (HR = 0.87; 95% CI, 0.66–1.15; *p* = 0.34) in mucosal melanoma [48].

On the other hand, it has been suggested that RT is an immune adjuvant boosting the antitumor immune response. At the same time, it is also possible that ITH has a radiosensitizing effect and increases the efficacy of radiotherapy [49,50]. In general, our MM study population is as large as the group that was identified in a pulled analysis of KEYNOTE-001, KEYNOTE-002 and KEYNOTE-006 trials [50]. We confirm the effect of RTH on PFS duration in MM patients, although larger prospective trials would be needed to validate our findings. In the case of MM, only international multicenter studies would be able to recruit a large number of patients due to the epidemiology of the disease.

## 4. Materials and Methods

### 4.1. Cohort

In this international retrospective study, we included consecutive patients with confirmed histopathological diagnosis of unresectable or metastatic MM. Patients must have been treated with anti-PD1 (nivolumab or pembrolizumab) or anti-CLTA-4 (ipilimumab) antibodies as a first or second-line treatment. Cases were recruited in five high-volume centers in Poland (two centers) and Italy (three centers) between July 2013 and August 2020.

### 4.2. Treatment

We analyzed the treatment sequence and response to systemic therapy, as well as factors that may influence on patients’ survival, including: patients’ age, sex, primary tumor site, the mutation in BRAF V600, tumor stage at the start of first-line treatment, number of metastatic sites (organs) at the start of first-line treatment, lactate dehydrogenase (LDH) levels at the start of first-line treatment, performance status at the start of first-line treatment and treatment sequence (ITH as a first or second-line). To define possible benefits from the addition of RT, we analyzed concurrent RT during anti-PD1 ITH and its parameters, regardless of the treatment line.

### 4.3. Response and Survival Analysis

The response was evaluated every 12 weeks in the contrast-enhanced CT scan of the primary tumor site, thorax, abdomen, and pelvis, and contrast-enhanced magnetic resonance imaging (MRI) for lesions in the central nervous system. Response to the treatment was assessed using response evaluation criteria in Solid Tumors 1.1 (RECIST). Objective response (OR) was defined as the sum of the number of patients who archived partial response (PR) or complete response (CR) as their best response. Disease control (DC) was defined as a sum of patients who archived stable disease (SD), PR or CR as their best response. Progression-free survival (PFS) was calculated from the start of treatment first dose to the disease progression, as assessed according to RECIST 1.1, or death. Patients who had at least stable disease (SD) at the last follow-up were censored. The overall survival (OS) was calculated from the start date of first-line treatment to the date of death. The OS from the start of anti-PD1 was also calculated. In both cases, patients alive at the last follow-up were censored.

### 4.4. Statistical Analysis

The continuous variables were summarized by median and interquantile range (IQR), while categorical variables were summarized by count and percentage of total cases. All point estimates were reported with a 95% confidence interval (CI) unless stated otherwise.

The Kaplan-Meier estimator with the log-rank test, as well as the Cox proportional hazard model, were used for the survival analysis. Fisher’s exact test was used to assess for independence between categorical variables.

All analyses were performed in the R language environment version 3.6.3 (The R Foundation for Statistical Computing) with abundant use of tidyverse and survminer packages [51,52,53,54]. A *p* ≤ 0.05 was deemed statistically significant.

### 4.5. Ethical Statement

This study was approved by Bioethical Committee at Maria Sklodowska-Curie National Research Institute of Oncology in Warsaw under the registration number 73/2018.

## 5. Conclusions

To our knowledge, this is the largest analysis of data to date for anti–PD-1 therapy combined with analysis of the radiation therapy impact on immunotherapy efficacy in mucosal melanoma. Anti-PD1 antibodies are an effective treatment in advanced MM. The addition of RT to ITH may be beneficial in the selected subgroup of MM patients.

## Figures and Tables

**Table 1:** The clinicopathological characteristics of patients.

Variable	Value	Number (Percentage)
*n*		82 (100)
Sex (%)	female	53 (64.6)
	male	29 (35.4)
Age (median [IQR])		67.50 [57.25, 75.75]
BRAF V600 mutation(%)	negative	77 (93.9)
	positive	5 (6.1)
Disease stage at the start of the treatment (%)	Localized, nonresectable disease	14 (17.1)
	M1a	6 (7.3)
	M1b	14 (17.1)
	M1c	43 (52.4)
	M1d	5 (6.1)
LDH at the start of the first-line (%)	Normal	43 (52.4)
	Over ULN	31 (37.8)
	Unknown	8 (9.8)
ECOG score at the start of first-line(%)	0	40 (48.8)
	1+	42 (51.2)
Localization (%)	Gastrointestinal system *	27 (33.0)
	Genitourinary tract	22 (26.8)
	Head and neck region	33 (40.2)
First-line treatment (%)	BRAFi+/-MEKi inhibitor	1 (1.2)
	Chemotherapy	5 (6.1)
	Anti-CTLA-4 antibody	13 (15.9)
	Anti-PD1 antibody	63 (76.8)
	Nivolumab	39 (47.6)
	Pembrolizumab	24 (29.2)
The best response to the first-line (%)	Complete response	4 (4.9)
	Partial response	15 (18.3)
	Stable disease	25 (30.5)
	Progressive disease	37 (45.1)
	Not evaluable	1 (1.2)
LDH at the start of the second-line (%)	Normal	14 (32.6)
	Over ULN	17 (39.5)
	Unknown	12 (27.9)
ECOG score at the start of second-line (%)	0	14 (32.6)
	1+	23 (53.4)
	Unknown	6 (14.0)
Second-line treatment (%)	Chemotherapy	6 (7.3)
	Anti-CTLA-4 antibody	21 (25.6)
	Imatinib	1 (1.2)
	Anti-PD1 antibody	15 (18.3)
	None	39 (47.6)
The best response to the second-line (%)	Complete response	2 (4.7)
	Partial response	1 (9.3)
	Stable disease	11 (25.6)
	Progressive disease	25 (58.1)
	Not evaluable	1 (1.2)
Brain metastases at the start of treatment (%)	Absent	77 (93.9)
	Present	5 (6.1)
Liver metastases at the start of treatment (%)	Absent	61 (74.3)
	Present	21 (25.6)
Number of organs involved at the start of treatment (%)	1	38 (46.3)
	2	23 (28.0)
	≥3	21 (25.6)
Radiotherapy during anti-PD1 treatment (%)	Not performed	65 (79.3)
	Performed	17 (20.7)

* comprised of 23 cases of anorectal mucosal melanoma (MM), two cases of esophageal MM, one case of stomach MM, and once case of MM in the gall bladder. IQR—interquartile range, LDH—lactate dehydrogenase, MEKi—MEK inhibitor, ULN—upper limit of normal, ECOG—Eastern Cooperative Oncology Group.

**Table 2:** Univariable Cox models for overall and progression-free survival. The 95% confidence intervals and *p* values are listed in brackets.

Variable	Value	HR for OS (Univariable)	HR for PFS (Univariable)
Age	Per one-year change	1.01 (0.98–1.03, *p* = 0.579)	1.01 (0.99–1.03, *p* = 0.436)
Sex	female	-	-
	male	1.83 (1.05–3.21, *p* = 0.034)	1.35 (0.81–2.26, *p* = 0.255)
BRAF V600 mutation	negative	-	-
	positive	0.62 (0.15–2.58, *p* = 0.516)	0.50 (0.15–1.58, *p* = 0.236)
Localization	Gastrointestinal system	-	-
	Genitourinary system	0.90 (0.43–1.88, *p* = 0.781)	1.28 (0.66–2.46, *p* = 0.462)
	Head and Neck Region	1.07 (0.57–2.02, *p* = 0.834)	1.39 (0.76–2.53, *p* = 0.290)
Disease stage at the start of the treatment	Localized, nonoperable disease	-	-
	M1a	3.11 (0.77–12.60, *p* = 0.112)	1.60 (0.54–4.68, *p* = 0.394)
	M1b	2.54 (0.82–7.85, *p* = 0.105)	1.61 (0.71–3.62, *p* = 0.252)
	M1c	1.89 (0.66–5.44, *p* = 0.238)	1.05 (0.51–2.15, *p* = 0.897)
	M1d	1.77 (0.39–8.00, *p* = 0.459)	1.57 (0.48–5.08, *p* = 0.455)
Number of organs involved at the start of treatment	1	-	-
	2	0.95 (0.48–1.88, *p* = 0.893)	1.26 (0.69–2.29, *p* = 0.453)
	3+	1.30 (0.65–2.58, *p* = 0.457)	1.54 (0.84–2.83, *p* = 0.162)
LDH at the start of the first-line	Normal	-	-
	overULN	2.11 (1.16–3.84, *p* = 0.014)	1.40 (0.82–2.37, *p* = 0.215)
	(Missing)	1.61 (0.67–3.89, *p* = 0.290)	1.45 (0.64–3.31, *p* = 0.375)
ECOG score at the start of first-line	0	-	-
	1+	1.19 (0.68–2.08, *p* = 0.553)	1.01 (0.61–1.65, *p* = 0.978)
The first line of treatment	PD1	-	-
	Other	0.90 (0.49–1.68, *p* = 0.747)	2.18 (1.26–3.76, *p* = 0.005)

## Supplementary Materials

The following are available online at https://www.mdpi.com/2072-6694/12/11/3131/s1. Supplement A table: Details regarding Radiotherapy during anti-PD1 treatment. Supplement B: Comparison of (A) progression-free survival and (B) overall survival between mucosal melanoma and Polish general melanoma cohort. Supplement C: PFS and OS in patients treated with radiotherapy with concomitant immunotherapy, chemotherapy of BRAFi/MEKi.
