# Supplementary figures and images for: Multimodal Treatment of Advanced Mucosal Melanoma in the Era of Modern Immunotherapy
Source: Cancers (Basel). 2020 Oct 26;12(11):3131. doi: 10.3390/cancers12113131 (PMC7692305; doi:10.3390/cancers12113131)

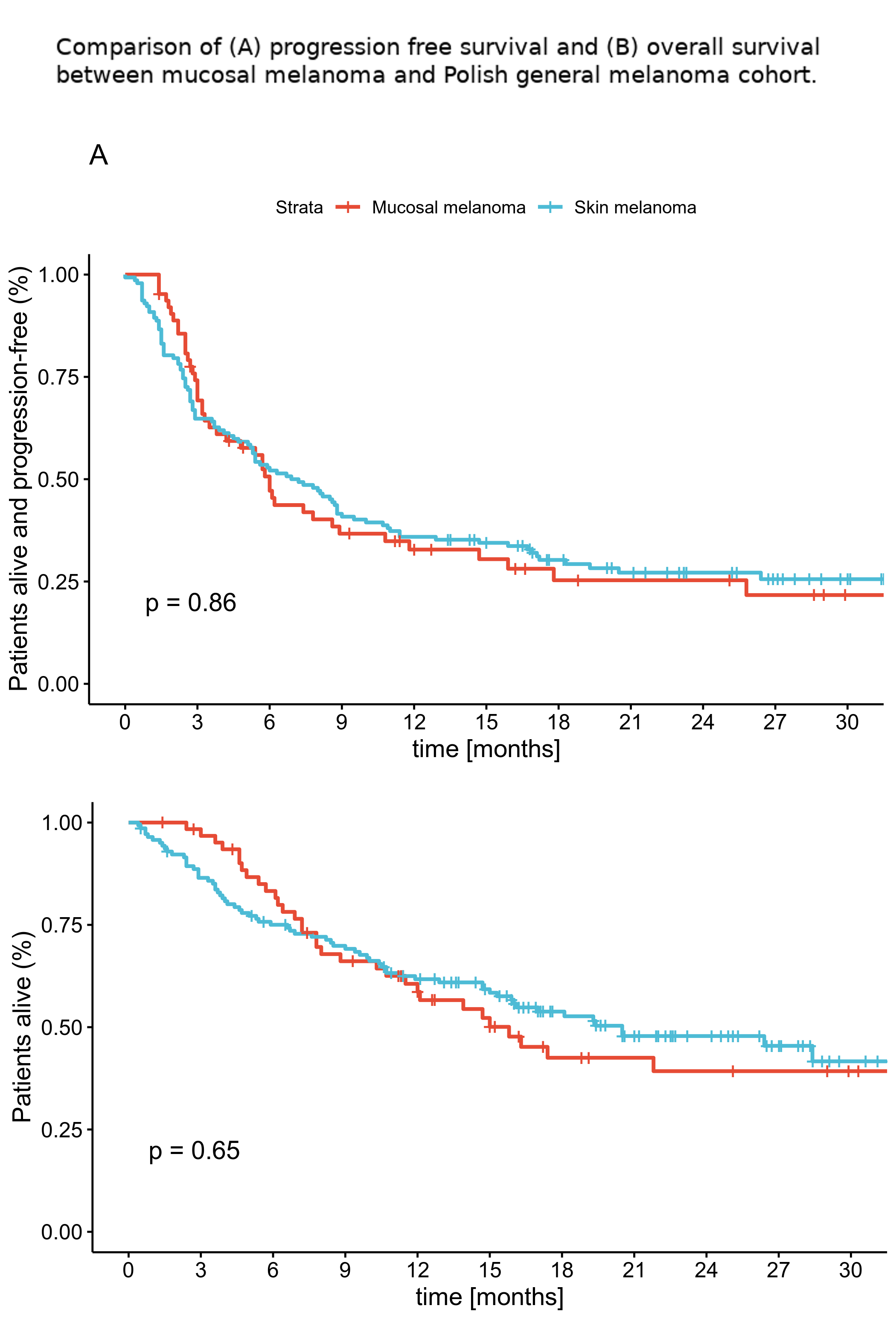

Supplement: Supplementary file 1 [file cancers-12-03131-s001.zip › cancers-931988-supplementary/SupplementB.png]

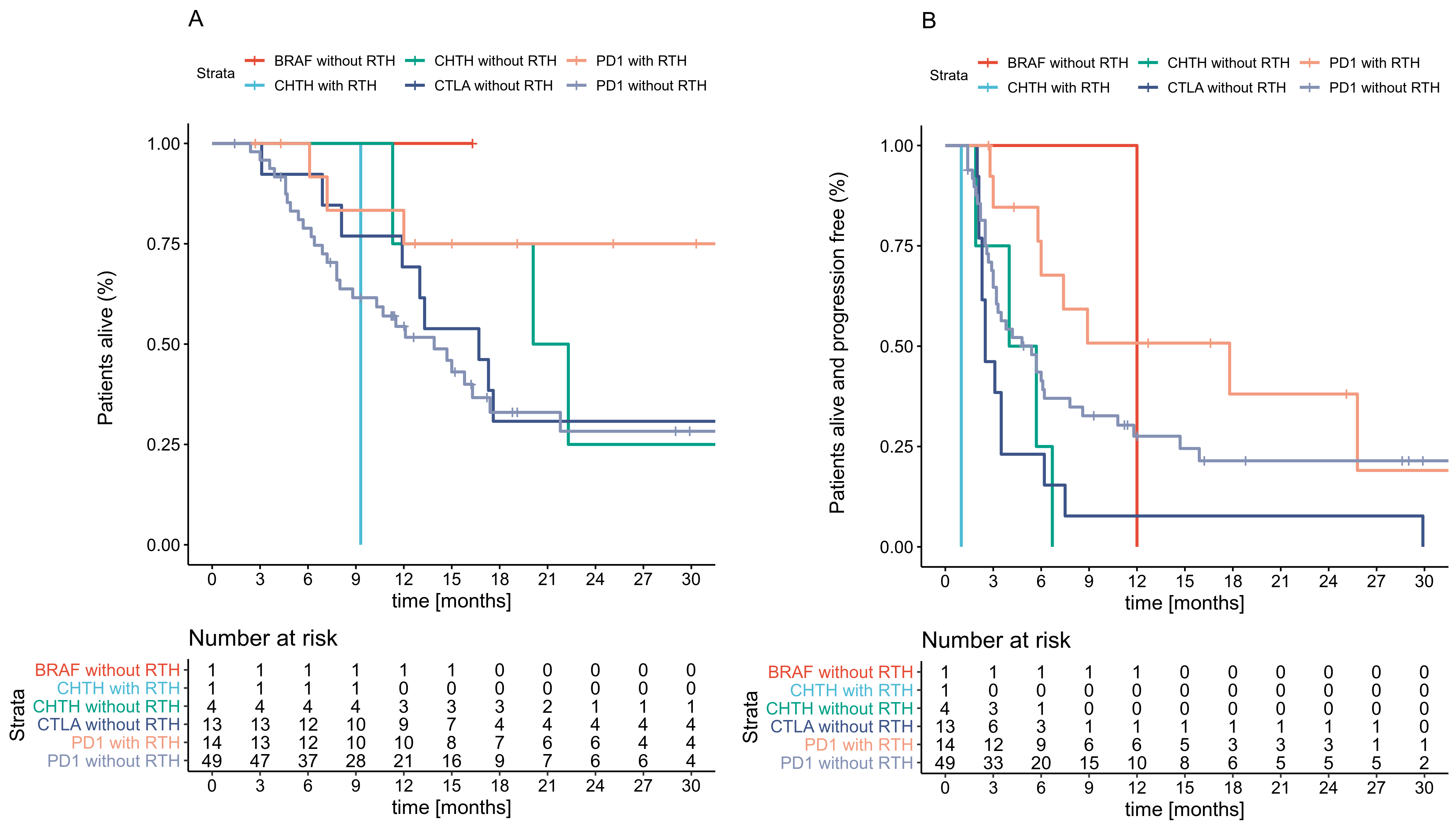

Supplement: Supplementary file 1 [file cancers-12-03131-s001.zip › cancers-931988-supplementary/supplementC.tiff]
